# Supplementary material for: Precise control of the interlayer twist angle in large scale MoS2 homostructures
Source: Nat Commun. 2020 May 1;11:2153. doi: 10.1038/s41467-020-16056-4 (PMC7195481; doi:10.1038/s41467-020-16056-4)
Supplement: Supplementary file 1 — Supplementary Information [file 41467_2020_16056_MOESM1_ESM.pdf]

Supplementary Information of

Precise control of the interlayer twist angle in large scale MoS_2 homostructures

Mengzhou Liao[1,2†], Zheng Wei[1,3†], Luojun Du[4†], Qinqin Wang[1,3], Jian Tang[1,3], Hua Yu[1,3], Fanfan Wu[1,3], Jiaojiao Zhao[1,3], Xiaozhi Xu[5], Bo Han[5], Kaihui Liu[5], Peng Gao[5], Tomas Polcar[2], Zhipei Sun[4,6], Dongxia Shi[1,3], Rong Yang[1,3,7*] and Guangyu Zhang[1,3,7*]

[1]*Beijing National Laboratory for Condensed Matter Physics and Institute of Physics, Chinese Academy of Sciences, Beijing 100190, China*
[2]*Faculty of Electrical Engineering, Czech Technical University in Prague, Technicka 2, 16627 Prague 6, Czech Republic*
[3] *School of Physical Sciences, University of Chinese Academy of Sciences, Beijing 100190, China*
[4] *Department of Electronics and Nanoengineering, Aalto University, Tietotie 3, FI-02150, Finland*
[5]*School of Physics, Peking University, Beijing 100871, China*
[6] *QTF Centre of Excellence, Department of Applied Physics, Aalto University, FI-00076 Aalto, Finland*
[7]*Songshan Lake Materials Laboratory, Dongguan, Guangdong 523808, China*

[†]Authors contributed equally to this work.

*Corresponding author. E-mail: ryang@iphy.ac.cn; gyzhang@iphy.ac.cn

Supplementary Notes 1: The uniformity of as-grown monolayer MoS_2 films.

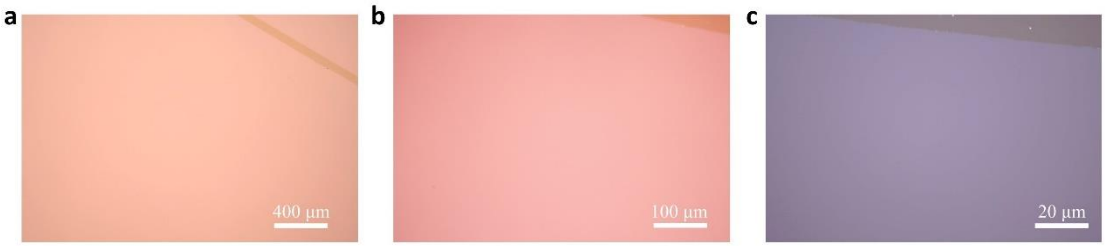

Supplementary Figure 1: Optical images of as-grown monolayer MoS_2 film on the sapphire substrate. a-c Image of as-grown MoS_2 monolayer surface under **a** 5x, **b** 20x and **c** 100x objective lens. We used a tweezer to scratch a line on it as a reference.

Supplementary Figure 1 shows the ultra-clean surface of our as-grown sample. There is no contamination or thick layer, indicating the high uniformity and quality of our sample.

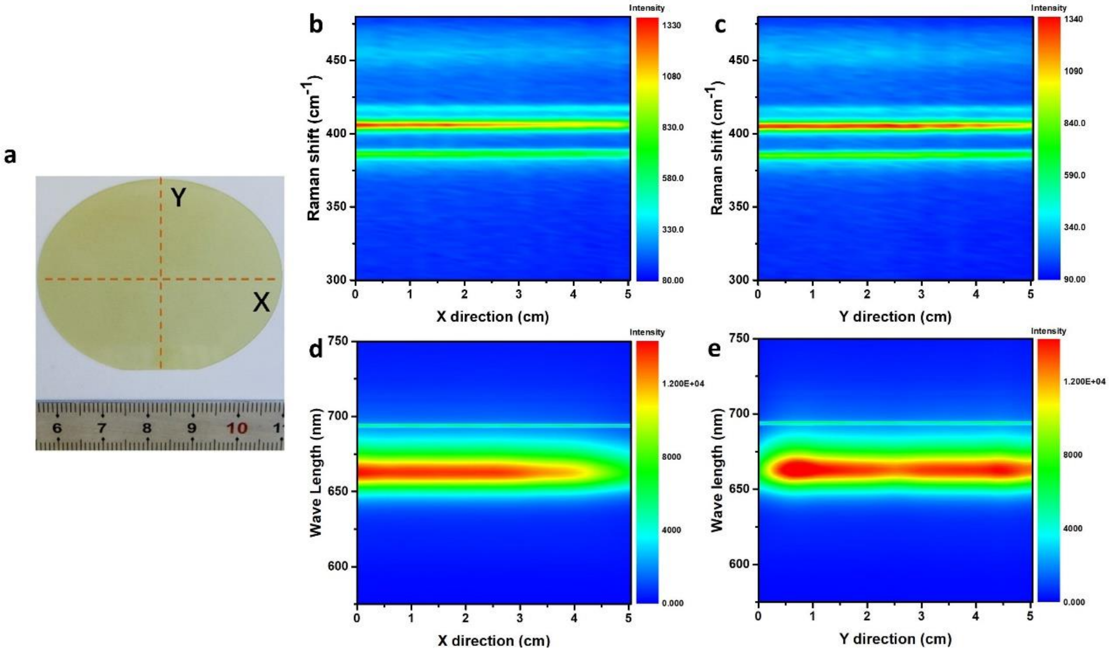

Supplementary Figure 2: Line scans Raman and PL spectra of an as-grown wafer. a A photo of as-grown wafer, red dash lines represent the line scan directions. **b-e** Line scan Raman spectra (**b** and **c**) and PL spectra (**d** and **e**) of X and Y directions. Each scan line contains 22 points. Source data are provided as a Source Data file.

From the Raman and PL line-scan spectra of an as-grown wafer in Supplementary Figure 2, we can see that both A_{1g}, E_{2g} and A exciton peaks have no shift over the whole wafer. PL intensity is uniform at the most area of the wafer except a very small part near the edge. These data show the wafer-scale uniformity of our monolayer MoS_2 films.

Supplementary Notes 2: Detail of the transfer process.

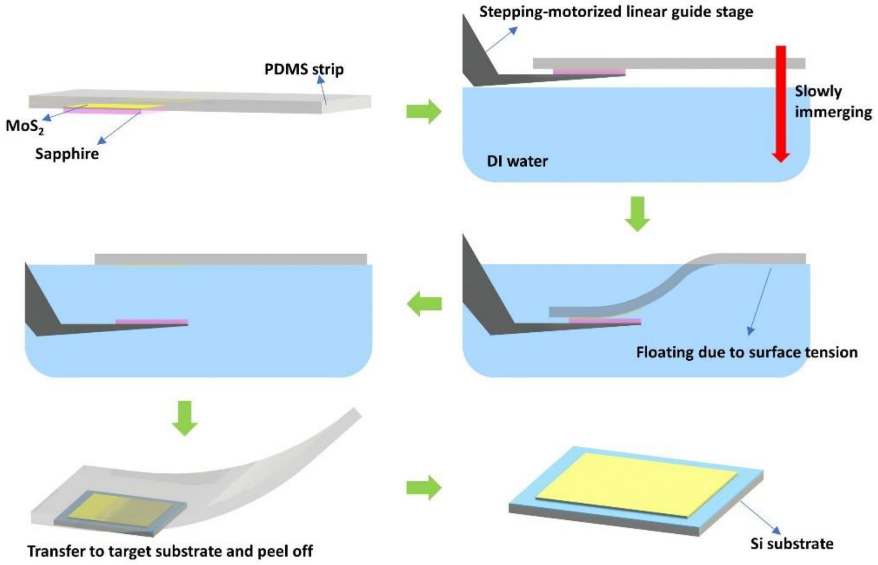

Supplementary Figure 3: Diagram of the transfer process. Polydimethylsiloxane (PDMS) are

used as transfer medium.

We firstly stamp a PDMS strip to MoS_2/sapphire slide and then stick this PDMS/MoS_2/sapphire slide to a step motor controlled linear guide stage. We slowly immerse the whole structure into DI water with a controlled speed of 1mm/min. Due to the surface of PDMS is hydrophobic, water surface tension will keep the PDMS strip float on the surface and offer a force to separate the PDMS and MoS_2/sapphire slide. As the wettability of MoS_2 and sapphire surface are different, the water will permeate into the interface through the slide edges, thus help to separate MoS_2 film from the sapphire substrate. After immersing the whole structure to a small depth about 5-6 mm, the MoS_2/PDMS will separate from sapphire and float on the water surface. Then, after dry MoS_2/PDMS under N_2 flow for a short time, we can stamp it to target substrates and peel off slowly to finish the transfer process.

Supplementary Notes 3: AFM images of all surfaces involved in transfer processes.

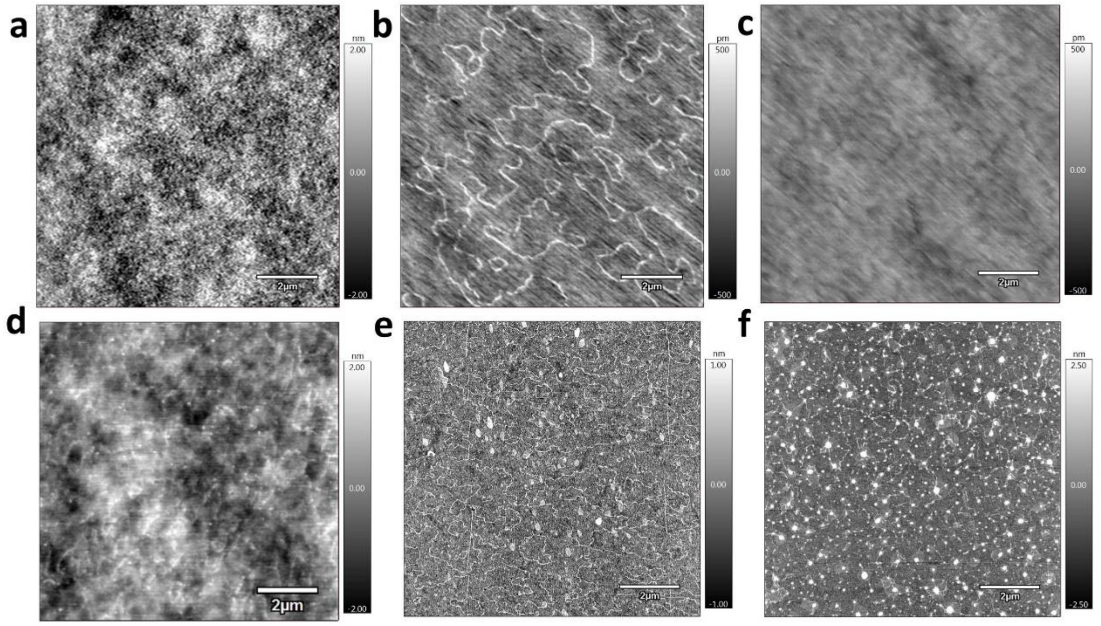

Supplementary Figure 4: AFM images of all surfaces involved in transfer processes. a PDMS surface. **b** As grown MoS_2/sapphire surface. **c** MoS_2/sapphire surface after stamping and peeling off by PDMS in air. **d** MoS_2/PDMS surface. **e** Surface of transferred monolayer MoS_2 on Si substrate with 300nm SiO_2. **f** Surface of transferred 30° bilayer MoS_2 on Si substrate with 300nm SiO_2. Source data are provided as a Source Data file.

Fig.S4 show the AFM images of all surfaces involved during the transfer process. From Supplementary Figure 4**a**, we can see the surface of PDMS is flat and clean, Ra=677.3pm, no contamination was observed. After PDMS stamping and peeling off in the air, nothing remains on MoS_2/sapphire sample surface, even previous contaminations on twin boundaries disappear (Supplementary Figure 4**b** and **c)**. Thus, PDMS will not induce any contaminations to MoS_2 surfaces (even can remove

contaminations), it is inert. Supplementary Figure 4**d** shows that after peeling off MoS_2 from sapphire by PDMS in DI water, the MoS_2/PDMS surface is still clean, which means no contamination will be induced by DI water. As illustrated in Supplementary Figure 4**e**, after annealing, the surface of transferred monolayer MoS_2 film is clean and flat. For the bilayer sample in Supplementary Figure 4**f**, most of the area is flat, only a few small bubbles exist (maximum 10%). Bilayer sample has more bubbles than monolayer is due to interlayer coupling between two MoS_2 layer is much stronger than that between MoS_2 and polycrystal SiO_2, which trapped gas cannot escape. These AFM images demonstrate our samples have both clean interfaces and surfaces.

Supplementary Notes 4: Precision of twist angle of transferred multilayer MoS_2 films.

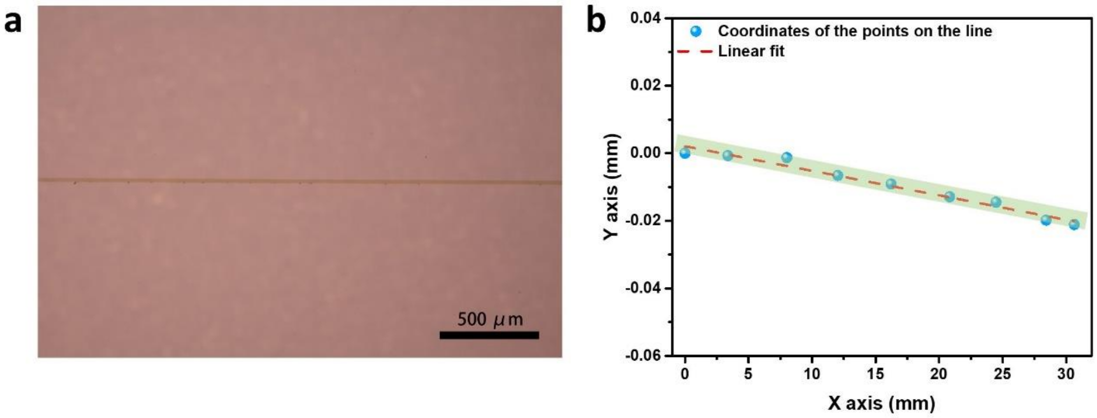

Supplementary Figure 5: Precision of linear guide. a The optical image of a line on MoS_2/sapphire surface scratched by Tungsten needle drove by the linear guide. **b** Coordinates of points on the line, measured by Nikon measuring microscope MM-200.

Here, precision and reproducibility of twist angles of transferred multilayer MoS_2 films are checked from two aspects. First is the method. The linear guide we used to cut the sapphire has maximum parallel misalignment about 20μm/100mm, which will induce a maximum 0.01° error. We also changed the diamond head to a tungsten needle and draw a line on MoS_2/sapphire sample surface to experimentally test the precision of the linear guide. Coordinates of points on the line were measured by Nikon Measuring Microscope MM-200. Form Supplementary Figure 5, the misalignment is below 0.005mm for a 30mm range, means a 0.0095° error, agree well with the precision of the linear guide. On the other hand, the rotational stage we used has the resolution 0.033° (1/30°), which can provide a relatively precise twist angle between two stack layers.

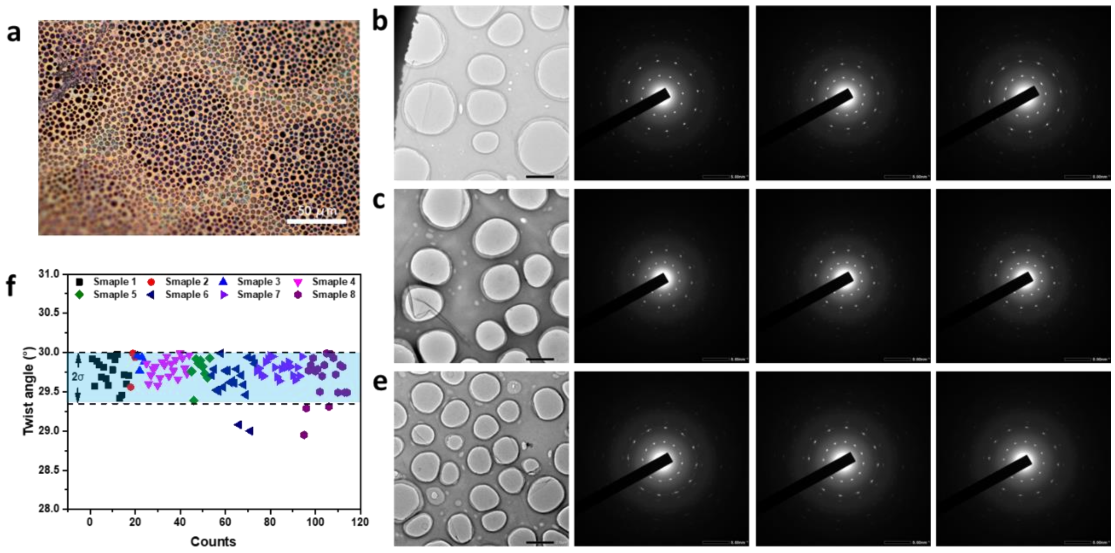

Supplementary Figure 6: Electron diffraction characterization of different regions on three 30° twisted bilayer samples. a Optical image of bilayer MoS_2 film on a TEM grid. **b-e** TEM images and electron diffraction patterns from different regions of three 30° twisted bilayer samples. **f** Twist angle distribution of different samples, σ is Standard Deviation. Source data are provided as a Source Data file.

Second is directly measure the twist angle of our bilayer samples by TEM electron diffraction from different regions. We tested eight 30° twisted bilayer samples. Supplementary Figure 6**a** shows a typical optical image of bilayer MoS_2 on a microgrid, transferred by standard wet process. Supplementary Figure 6**b-e** are TEM images and electron diffraction patterns from different regions of three 30° twisted bilayer samples. Supplementary Figure 6**f** is the Twist angle distribution of different samples. From the statistics, we obtain the Standard Deviation (Std Dev) of the twist angle is 0.327°. From some electron diffraction, we can see some patterns are not perfectly symmetric. So we think the strain induced during the TEM sample preparation, misalignment between the electron beam and the c axis of samples, and wrinkles also contributed to the error.

Supplementary Notes 5: Indirect bandgap peak position mapping.

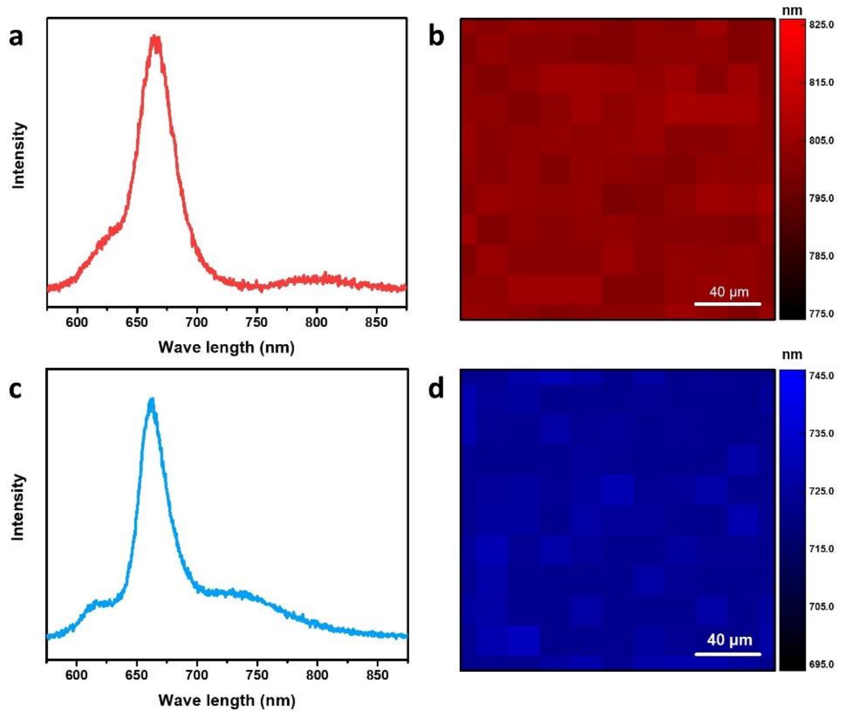

Supplementary Figure 7: Indirect bandgap peak position mapping of another pair of 0°/30° samples. a-b Single PL spectrum **a** and indirect bandgap peak position mapping **b** of 0° bilayer sample. **c-d** Single PL spectrum **c** and indirect bandgap peak position mapping **d** of 0° bilayer sample. Source data are provided as a Source Data file.

We did 200×200 μm^2 mappings again on new 0° and 30° samples. Supplementary Figure 7**a-b** and **c-d** are single spectra and mapping of 0° and 30° twisted bilayers. We can see the positions of indirect bandgap peaks are still homogeneous. The indirect bandgap peaks' position of our new sample is a little blue shift compare to which in the main text, but the tendency is the same. This may occur due to different doping levels of different processes of growth (Sapphire wafers offered by two companies).

Supplementary Notes 6: More details about PL spectra.

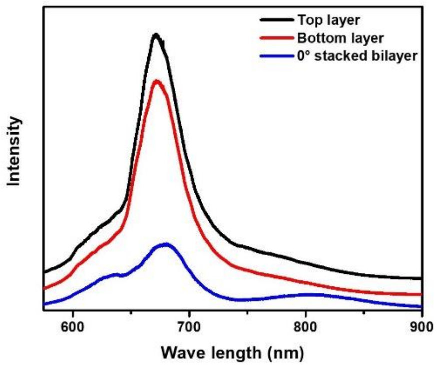

Supplementary Figure 8: PL spectra of the top layer, bottom layer, and stacked bilayer. Source data are provided as a Source Data file.

We measured the PL spectra of a 0 °stacked bilayer sample. From Supplementary Figure 8, the PL spectra of top and bottom layers are almost identical. So, we can safely say our transfer method would not damage MoS_2 films.

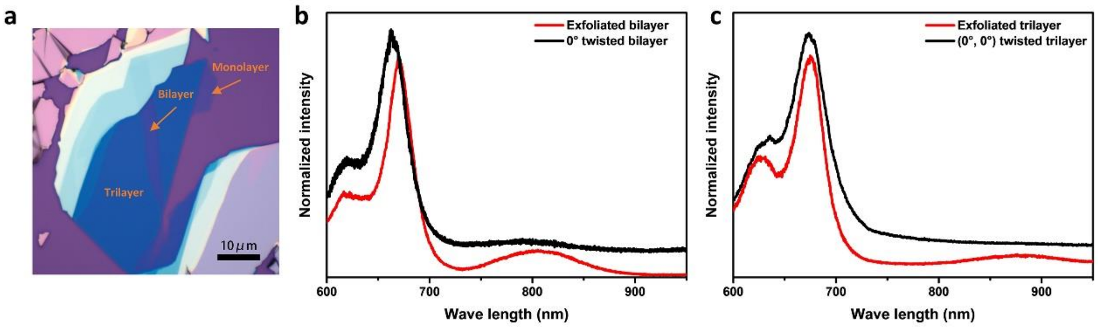

Supplementary Figure 9: PL spectra of natural and transferred bilayer and trilayer MoS_2 films. a Optical image of an exfoliated MoS_2 flake. **b-c** PL spectra of natural and transferred bilayer **b** and trilayer **c** MoS_2 films. Source data are provided as a Source Data file.

The comparison of exfoliated MoS_2 and stacked MoS_2 is as Supplementary Figure 9. From which we can see that the artificial and natural bilayer MoS_2 films have similar peak positions of B, A, and indirect bandgap excitons. The only difference is the intensity.

Supplementary Notes 7: I/Vg curves of bilayer devices and mobility calculation.

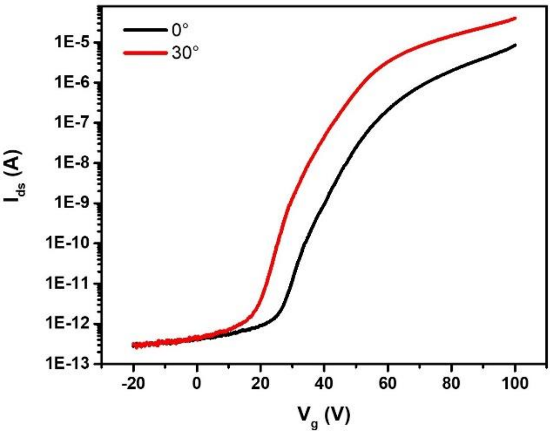

Supplementary Figure 10: Electrical transfer curves of a typical 30 °/0 °twisted bilayer MoS_2 FETs. Source data are provided as a Source Data file.

The mobility of a device was calculated by the formula $\mu = \frac{L}{W}\frac{d}{\varepsilon_0\varepsilon_r}\frac{1}{V_{sd}}\frac{\partial I_{sd}}{\partial V_g}$. Where in one device as show in Fig. 5**a**, L=5, 10, 15, 20, 25, 30, 35μm respectively, and W=5-35μm for different device array. d=300nm is the thickness of SiO_2, $\varepsilon_0 \approx$ 8. 85×10^{-12} F m^{-1} and ε_r ≈3.9 are the vacuum permittivity and relative permittivity of

SiO_2, respectively. The $\frac{\partial I_{sd}}{\partial V_g}$ was calculated from the I/V_g curves. We tested 30 devices with different channel lengths and calculated the channel mobilities by transfer-length-method.
